# Impact of dementia and drug compliance on patients with acute myocardial infarction

**DOI:** 10.1002/clc.24091

**Published:** 2023-07-24

**Authors:** Wonjae Lee, Si‐Hyuck Kang, Sun‐Hwa Kim, Seung‐Yeon Lee, Woojae Myung, Ki‐Hyun Jheon, Chang‐Hwan Yoon, Jung‐Won Suh, Tae‐Jin Youn, In‐Ho Chae

**Affiliations:** ^1^ Department of Internal Medicine, Division of Cardiology Cardiovascular Center, Seoul National University Bundang Hospital Seongnam‐si Gyeonggi‐do Korea; ^2^ International Healthcare Center Seoul National University Bundang Hospital Seongnam‐si Gyeonggi‐do Korea; ^3^ Department of Psychiatry Seoul National University Bundang Hospital Seongnam‐si Gyeonggi‐do Korea

**Keywords:** acute myocardial infarction, all‐cause mortality, cognitive disorder, medication adherence, Republic of Korea

## Abstract

**Background:**

In South Korea, the number of people with dementia is rising at a worrisome rate, and many of them also have acute myocardial infarction (AMI), a disease with a high mortality rate.

**Hypothesis:**

We speculated that dementia and drug compliance have significant impact on the mortality of patients with AMI.

**Methods:**

The study derived data from the National Health Insurance Service‐Senior for a retrospective cohort study. The total number of patients diagnosed with AMI for the first time between 2007 and 2013 was 16 835, among whom 2021 had dementia. Medication possession ratio (MPR) was used to assess medication adherence.

**Results:**

AMI patients with dementia had unfavorable baseline characteristics; they had significantly higher risk of all‐cause mortality (hazard ratio [HR]: 2.49; 95% confidence interval [CI]: 2.34−2.66; *p* < .001) and lower MPR (aspirin: 21.9% vs. 42.8%; *p* < .001). AMI patients were stratified by presence of dementia and medication adherence, and the survival rate was the highest among those with no dementia and good adherence, followed by those with no dementia and poor adherence, those with dementia and good adherence, and those with dementia and poor adherence. The multivariable analysis revealed that dementia (HR: 1.64; 95% CI: 1.53−1.75; *p* < .001) and poor adherence to medication (HR: 1.60; 95% CI: 1.49−1.71; *p* < .001) had a significant association with all‐cause mortality in AMI patients.

**Conclusions:**

AMI patients with dementia have a higher mortality rate. Their prognosis is negatively affected by their poorer medication adherence than patients without dementia.

## Graphical abstract

Based on the the National Health Insurance Service‐Senior Cohort, dementia (hazard ratio [HR]: 1.64; 95% confidence interval [CI]: 1.53−1.75; *p* < .001) and poor adherence to medication (HR: 1.60; 95% CI: 1.49−1.71; *p* < .001) had a significant association with all‐cause mortality in acute myocardial infarction patients.

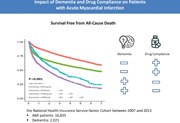

## 1 INTRODUCTION

Worldwide, the number of people living with dementia is increasing rapidly and may reach 152 million by 2050.^1^ In older adults, dementia increases the risk of death by 1.8−6.3 times.^2^, ^3^, ^4^ A study that compared populations of older adults with dementia and those with normal cognitive function reported that the former population faces a risk of death that is approximately 8 times higher.^5^ Reports have indicated that the average life expectancy for people with dementia is 3−9 years after disease onset.^3^, ^6^ However, the range is widely distributed, and people who were physically healthy when they were diagnosed with dementia are known to live much longer. Experts speculate that the early death among patients with dementia is attributable to a delayed diagnosis of comorbidity, which consequently leads to suboptimal care and management.^7^, ^8^, ^9^ In fact, cardiovascular disease is one of the main causes of death among people with dementia.^10^, ^11^ However, little is known about how dementia affects the prognosis and care of patients with cardiovascular disease, particularly those with acute myocardial infarction (AMI). We sought to investigate the association between dementia and all‐cause mortality in patients with AMI and identify the factors—such as adherence to medication—that affect prognosis.

## 2 METHODS

### 2.1 Data source

Data were derived from the National Health Insurance Service (NHIS)‐Senior, the database of a sample cohort of all older adults aged ≥60 (5.5 million) in 2002 in South Korea.^12^ Of these, 558 147 people were selected by a simple random sampling method. NHIS‐Senior contains the diagnosis codes of the International Statistical Classification of Diseases and Related Health Problems; Tenth Revision (ICD‐10); and claims information, including prescriptions, sociodemographic and socioeconomic information, and insurance status. The study protocol was approved by the Institutional Review Board of Seoul National University Bundang Hospital (IRB No. X‐1911‐577‐904), and the study was conducted in accordance with the Helsinki Declaration. The need for informed consent from study participants was waived.

### 2.2 Study population

**Figure 1:**
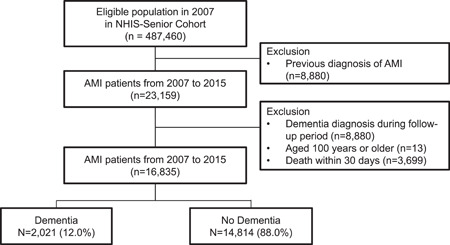
Flow chart of the classification of the study population. AMI, acute myocardial infarction; NHIS, National Health Insurance Service.

We extracted data on patients diagnosed with AMI during hospital admission from 2007 to 2015. We used a 5‐year wash‐out period (between 2002 and 2006) to ensure that the sample had no previous diagnoses of AMI. We identified 23 159 newly diagnosed patients with AMI (Figure 1). Those with a diagnosis of dementia before and within 30 days of index admission for AMI were classified as the dementia group. Patients who were diagnosed with dementia during the follow‐up period (*n* = 2612), aged 100 years or older (*n* = 13), or had died within 30 days of AMI diagnosis (*n* = 3699) were excluded from the analysis.

AMI was defined based on the presence of symptoms characteristic of ICD‐10 codes I21 and I22 during the index admission.^13^ For dementia, the diagnosis was confirmed if ICD‐10 codes F00, F01, F02, F03, and G30 were noted during an admission or observed at least twice in the outpatient clinic. This definition was validated in prior studies.^14^, ^15^ To assess medication adherence, we utilized the medication possession ratio (MPR), defined as the number of days' medication supplied divided by the number of days in a time period.^16^ We calculated the average of MPR of the first, second, and third years to compare the no‐dementia and dementia groups. To further stratify patients, we divided patients into two groups (good MPR and poor MPR) with the cut‐off at 50% in the first year. The primary endpoint was all‐cause mortality.

### 2.3 Statistical analysis

Continuous variables are expressed as means ± standard deviation, whereas categorical variables are expressed as proportions. We used student's *t*‐tests and *χ* ^2^ tests to evaluate group differences in continuous and categorical variables, respectively. Kaplan−Meier curves were used to visualize and identify the primary endpoint of patients with or without dementia, and the difference was evaluated by the log‐rank test, also used for the pairwise comparison of subgroups.

The Cox proportional hazards model was constructed to adjust all variables collected for baseline characteristics and MPR. The adjusted covariates were age, sex, hypertension, diabetes, dyslipidemia, chronic kidney disease, end‐stage renal disease, peripheral arterial occlusive disease, chronic obstructive pulmonary disease, liver disease, malignancy, income levels, discharge medications, and MPR of aspirin in the first year. The results were expressed in terms of a hazard ratio (HR) and the corresponding 95% confidence interval (CI). We then performed stepwise regression under Akaike's information criterion to determine the appropriate multivariate model. All reported *p*‐values were two‐tailed, and *p* ≤ .050 indicated statistical significance. All statistical analyses were performed using R Statistical Software/environment (version 3.4.3; The R foundation for Statistical Computing).

## 3 RESULTS

Among the 16 835 subjects included in this study, 2021 (12.0%) had dementia (Table 1). The median follow‐up duration was 2.7 years (interquartile range: 0.8−5.4 years). We found a significant difference in baseline characteristics between AMI patients with and without dementia: the former were older and likely to be female, and had more comorbidities, whereas the latter had higher educational levels and were more likely to be discharged with standard medication.

**Table 1:** Baseline characteristics of the participants.

	Overall population		
	Dementia (−) (*n* = 14 814)	Dementia (+) (*n* = 2021)	*p*
Demographics			
Age (years)	73.1 ± 6.1	76.6 ± 6.4	<.001
Male sex (%)	6624 (44.7)	617 (30.5)	<.001
Comorbidities (%)			
Hypertension	12 930 (87.3)	1877 (92.9)	<.001
Diabetes	4394 (29.7)	653 (32.3)	.016
Dyslipidemia	11 685 (78.9)	1555 (76.9)	.050
Heart failure	7893 (53.3)	1319 (65.3)	<.001
CKD or ESRD	262 (1.8)	83 (4.1)	<.001
PAOD	5867 (39.6)	920 (45.5)	<.001
COPD	5112 (34.5)	770 (38.1)	.002
Liver disease	639 (4.3)	96 (4.8)	.399
Malignancy	3859 (26.0)	533 (26.4)	.777
Income levels (%)			<.001
Low	3884 (26.2)	716 (35.4)	
Middle	5769 (38.9)	656 (32.5)	
High	5161 (34.8)	649 (32.1)	
Medication at discharge			
ACE inhibitor or ARB (%)	5193 (35.1)	323 (16.0)	<.001
Beta blocker (%)	4079 (27.5)	202 (10.0)	<.001
CCB (%)	1364 (9.2)	151 (7.5)	.012
Statin (%)	4754 (32.1)	259 (12.8)	<.001
Aspirin (%)	6467 (43.7)	388 (19.2)	<.001
P2Y12 inhibitor (%)	5128 (34.6)	300 (14.8)	<.001

*Note*: Data are presented as mean ± SD.

Abbreviations: ACE, angiotensin converting enzyme; ARB, angiotensin receptor blocker; CCB, calcium channel blocker; CKD, chronic kidney disease; COPD, chronic obstructive pulmonary disease; ESRD, end stage renal disease; PAOD, peripheral artery obstructive disease.

**Figure 2:**
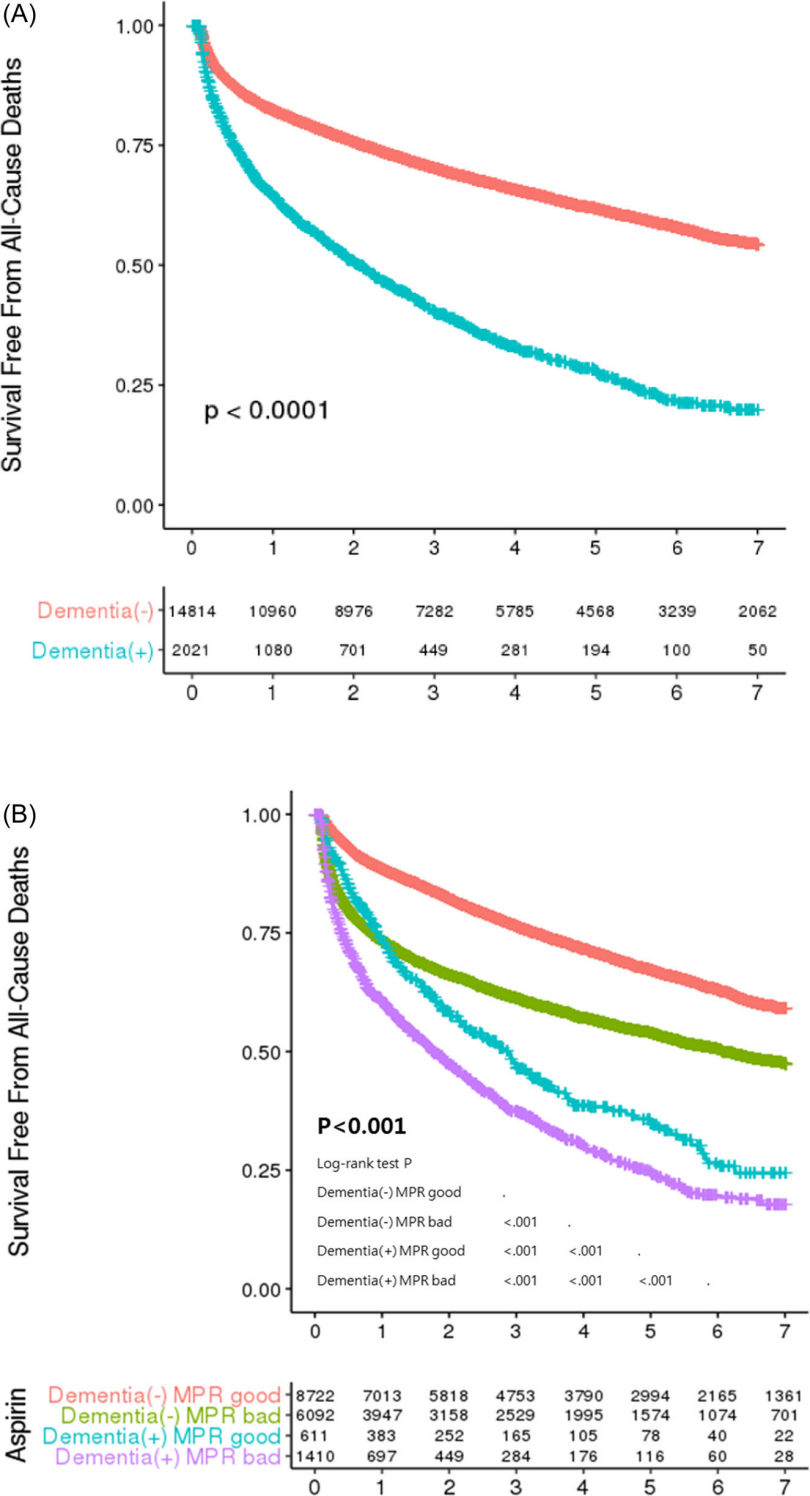
Kaplan−Meier curves for comparison of AMI patients according to the presence of (A) dementia and (B) dementia and MPR. AMI, acute myocardial infarction; MPR, medication possession ratio.

During the follow‐up period, 58.1% and 34.8% of AMI patients with and without dementia, respectively, died. Patients with dementia had a significantly higher risk of all‐cause mortality (HR: 2.49; 95% CI: 2.34−2.66; *p* < .001), as shown in Figure 2A. The Kaplan−Meier curves diverged from the beginning, and the difference became more marked in the follow‐up period.

We evaluated MPR in the first 3 years after the index admission. MPR of patients with and without dementia, considering all medications, continued to decrease in the second and third years (Table 2). When comparing the MPRs of patients with and without dementia, we found a significant difference: the MPR of aspirin was only 21.9% in patients with dementia but 42.8% in those without (*p* < .001) during the first year. Similarly, the MPR of statin was 29.1% versus 56.9% in patients with and without dementia, respectively (*p* < .001).

**Table 2:** Trend of medication possession ratio over time.

	Overall population		
	Dementia (−) (*n* = 14 814)	Dementia (+) (*n* = 2021)	*p*
MPR (first year)			
ACE inhibitor or ARB	55.7 ± 47.4	28.0 ± 43.4	<.001
Beta blocker	41.9 ± 47.6	16.9 ± 36.7	<.001
CCB	31.3 ± 44.4	18.4 ± 37.3	<.001
Statin	47.3 ± 48.1	19.6 ± 38.8	<.001
Aspirin	56.9 ± 47.0	29.1 ± 43.9	<.001
P2Y12 inhibitor	42.8 ± 47.9	21.9 ± 40.4	<.001
MPR (second year)			
ACE inhibitor or ARB	45.0 ± 47.8	19.7 ± 38.7	<.001
Beta blocker	31.7 ± 45.0	10.6 ± 30.1	<.001
CCB	23.5 ± 40.9	14.5 ± 34.2	<.001
Statin	41.2 ± 47.4	14.9 ± 34.9	<.001
Aspirin	45.3 ± 47.7	20.9 ± 39.5	<.001
P2Y12 inhibitor	33.4 ± 45.7	15.0 ± 34.9	<.001
MPR (third year)			
ACE inhibitor or ARB	42.4 ± 47.7	17.2 ± 37.1	<.001
Beta blocker	29.3 ± 44.4	9.0 ± 28.2	<.001
CCB	22.5 ± 40.5	11.9 ± 31.6	<.001
Statin	40.4 ± 47.5	13.9 ± 34.0	<.001
Aspirin	40.6 ± 47.5	18.1 ± 37.8	<.001
P2Y12 inhibitor	28.5 ± 44.0	13.3 ± 33.6	<.001

*Note*: Data are presented as mean ± SD.

Abbreviations: ACE, angiotensin converting enzyme; ARB, angiotensin receptor blocker; CCB, calcium channel blocker; MPR, medication possession ratio.

Figure 2B shows how medication adherence impacted the survival of AMI patients with and without dementia. Based on the MPR of aspirin of the first year, the patients were divided into four subgroups. The survival rates at 7 years for the four subgroups—no dementia with good adherence, no dementia with poor adherence, dementia with good adherence, and dementia with poor adherence—were 82.2%, 75.6%, 52.6%, and 40.9%, respectively (*p* < .001). The differences among the subgroups were all significant. The dementia with good adherence group tended to have a better survival rate within 1 year, compared with the no dementia with poor adherence group, but the survival curves for both groups converged around 1 year. The Kaplan−Meier curves of other medications demonstrated an almost identical trend (Supporting Information: Figure S1).

To account for the significant difference in baseline characteristics between AMI patients with and without dementia, we conducted a multivariate analysis. All variables had a significant association with all‐cause mortality in the univariate analysis (Table 3). Dementia had an HR of 2.49 (CI: 2.34−2.66); poor adherence of aspirin had an HR of 1.83 (CI: 1.74−1.92). After adjustment with covariates, dementia and poor adherence continued to be significantly associated with all‐cause mortality in AMI patients, with HRs of 1.64 (CI: 1.53−1.75) and 1.60 (CI: 1.49−1.71), respectively.

**Table 3:** Cox proportional hazards analysis of covariates for all‐cause mortality.

	Univariable			Multivariable		
	HR	CI	*p*	HR	CI	*p*
Dementia (+)	2.49	2.34−2.66	<.001	1.64	1.53−1.75	<.001
Age	1.08	1.08−1.09	<.001	1.08	1.07−1.08	<.001
Female sex	0.78	0.74−0.82	<.001	0.60	0.57−0.63	<.001
Hypertension	1.26	1.17−1.37	<.001	1.27	1.16−1.38	<.001
Diabetes	1.16	1.10−1.22	<.001	1.40	1.33−1.48	<.001
Dyslipidemia	0.70	0.66−0.74	<.001	0.86	0.81−1.48	<.001
Heart failure	1.48	1.40−1.55	<.001	1.38	1.31−1.46	<.001
CKD or ESRD	1.89	1.61−2.23	<.001	1.67	1.41−1.97	<.001
PAOD	0.92	0.87−0.97	.001	0.93	0.88−0.98	.008
COPD	1.42	1.35−1.50	<.001	1.11	1.06−1.17	<.001
Liver disease	1.33	1.20−1.49	<.001	1.08	0.97−1.21	.167
Malignancy	1.48	1.41−1.57	<.001	1.26	1.19−1.33	<.001
Income levels						
Middle	0.86	0.81−0.91	<.001	0.95	0.89−1.01	.092
High	0.84	0.79−0.89	<.001	0.92	0.86−98	.006
Medication at discharge						
ACE inhibitor or ARB	0.62	0.59−0.66	<.001	0.83	0.77−0.89	<.001
Beta blocker	0.59	0.55−0.63	<.001	0.89	0.83−0.96	.004
CCB	0.83	0.76−0.91	<.001	0.83	0.76−0.91	<.001
Statin	0.50	0.47−0.554	<.001	0.74	0.69−0.80	<.001
Aspirin	0.63	0.60−0.66	<.001	1.27	1.17−1.39	<.001
P2Y12 inhibitor	0.60	0.56−0.63	<.001	0.95	0.87−1.03	.189
MPR (first year)						
Aspirin (<50%)	1.83	1.74−1.92	<.001	1.60	1.49−1.71	<.001

Abbreviations: ACE, angiotensin converting enzyme; ARB, angiotensin receptor blocker; CCB, calcium channel blocker; CI, confidence interval; CKD, chronic kidney disease; COPD, chronic obstructive pulmonary disease; ESRD, end stage renal disease; HR, hazard ratio; MPR, medication possession ratio; PAOD, peripheral artery obstructive disease.

## 4 DISCUSSION

AMI patients with dementia had a substantially higher risk of death compared with those without dementia. While dementia was associated with poor drug compliance, dementia and poor drug compliance were shown to be independent risk factors for mortality. The fact that AMI patients with dementia had unfavorable baseline factors could not fully explain the increased risk of mortality in the dementia group.

Studies have focused on evaluating cardiovascular disease as the cause of cognitive decline among patients and finding treatments that may prevent dementia in this population.^16^, ^17^ A prospective study on post‐menopausal women revealed that patients with cardiovascular disease have a 29% higher risk of developing mild cognitive dysfunction or dementia.^18^ A meta‐analysis revealed that coronary heart disease is associated with cognitive decline or dementia.^17^ In low‐risk patients with atrial fibrillation, oral anticoagulants may prevent dementia and stroke.^19^ Some studies have reported the association of statin with cognitive decline, although more recent studies favor the role of statin in dementia and cardiovascular disease.^20^, ^21^, ^22^, ^23^ In this work, we opted to focus on investigating how dementia impacts the life of AMI patients.

AMI is a disease with a high mortality rate. Dementia is also known to increase the risk of death in the general population.^24^ However, data are scarce on the prognosis of patients with dementia also diagnosed with AMI. To our knowledge, our work is the first population‐based study to investigate how dementia affects the prognosis of patients with AMI. We compared the survival rate of patients with and without dementia after they were discharged with a diagnosis of AMI, and demonstrated that the probability of all‐cause mortality reached almost 50% after 2 years in AMI patients with dementia, which is double that among patients without dementia. Patients with dementia have more comorbidities and, more importantly, often have a poorer socioeconomic status, which our study confirmed.^25^, ^26^, ^27^ Socioeconomic inequality leads to poor people having fewer years of formal education and a higher risk for dementia.^28^ This group also tends to have a late diagnosis and, often, no access to proper caregiving because of financial problems. Thus, income and education inequalities may lead to suboptimal care, resulting in poor prognosis.

This study offers important clinical insights into dementia patient care for older adults. While there is no proven, robust way to prevent or treat dementia,^29^ providing optimal care can be a feasible and potent measure to improve the prognosis of patients with dementia. In fact, life expectancy varies considerably among those diagnosed with dementia.^30^ Our findings indicated that patients with dementia may live longer and have healthier lives when their disease is managed and treated properly. A previous study found that low socioeconomic status and poor adherence to antihypertensive medication are associated with higher risks for developing cardiovascular disease.^31^ Given that people with dementia typically endure such conditions, they are likely to face a higher risk of cardiovascular disease. Additionally, guideline‐directed medical treatment and optimal adherence to medication is highly critical for AMI.^32^ Our study demonstrated that patients with dementia had much lower medication adherence compared with those without dementia, and this state worsened over time. While poorer adherence to medication led to worse clinical outcomes, an interesting finding was that patients with dementia who had good drug compliance had a significantly better clinical course than those without dementia who had poor drug compliance in the 1 year after AMI diagnosis.

## 5 LIMITATIONS

To obtain a homogenous set of a newly diagnosed AMI cohort, we applied a 5‐year wash‐out period. However, our data lacked sufficient information on AMI subtype or etiology, such as thrombotic and type II myocardial infarctions. Another limitation was that the duration of dementia differed across patients, given that we included all patients with a diagnosis of dementia before and within 30 days of index admission for AMI. As length bias has a profound effect on mortality risk and incident dementia (new diagnosis of dementia on follow‐up) has a higher mortality risk, our study could have underestimated the risks.^5^ We also assumed the medication adherence based on MPR values.^16^ Although MPR is a well‐validated proxy for drug compliance, its use remains a limitation. As our study is retrospective, prospective research is warranted to validate the effect of drug compliance and dementia in AMI patients.

## 6 SUMMARY

AMI patients with dementia experienced a grave clinical course. They had worse clinical features, including medication adherence. Dementia and poor medication adherence had a significant impact on the prognosis of AMI patients.

## CONFLICT OF INTEREST STATEMENT

The authors declare no conflict of interest.

## Supporting information


**Figure S1**. Kaplan‐Meier curves for comparison of AMI patients according to the presence of dementia and MPR of standard medications. Note: AMI = acute myocardial infarction, MPR = medication possession ratio, CCB = calcium channel blocker, ACE = angiotensin converting enzyme inhibitors, ARB = angiotensin receptor blockers, P2Y12 = P2Y_12_ inhibitors.

## Data Availability

The data that support the findings of this study are available from the National Health Insurance Service, with the permission of the authorizing committee. Restrictions apply to the availability of the data, which were used under license for this study.

## References

[1] Patterson C . World Alzheimer Report 2018. Alzheimer's Disease International; 2018.

[2] Helmer C . Mortality with dementia: results from a French prospective community‐based cohort. Am J Epidemiol. 2001;154:642‐648. 10.1093/aje/154.7.642 11581098

[3] Fitzpatrick AL , Kuller LH , Lopez OL , Kawas CH , Jagust W . Survival following dementia onset: Alzheimer's disease and vascular dementia. J Neurol Sci. 2005;229‐230:43‐49. 10.1016/j.jns.2004.11.022 15760618

[4] Park JE , Lee JY , Suh GH , Kim BS , Cho MJ . Mortality rates and predictors in community‐dwelling elderly individuals with cognitive impairment: an eight‐year follow‐up after initial assessment. Int Psychogeriatr. 2014;26:1295‐1304. 10.1017/s1041610214000556 24965360

[5] Bae JB , Han JW , Kwak KP , et al. Is dementia more fatal than previously estimated? A population‐based prospective cohort study. Aging Dis. 2019;10:1‐11. 10.14336/AD.2018.0123 30705763 PMC6345342

[6] Xie J , Brayne C , Matthews FE . Survival times in people with dementia: analysis from population based cohort study with 14 year follow‐up. BMJ. 2008;336:258‐262. 10.1136/bmj.39433.616678.25 18187696 PMC2223023

[7] Löppönen MK , Isoaho RE , Räihä IJ , et al. Undiagnosed diseases in patients with dementia—a potential target group for intervention. Dementia Geriatr Cognit Disord. 2004;18:321‐329. 10.1159/000080126 15305110

[8] Bauer K , Schwarzkopf L , Graessel E , Holle R . A claims data‐based comparison of comorbidity in individuals with and without dementia. BMC Geriatr. 2014;14:10. 10.1186/1471-2318-14-10 24472217 PMC3909381

[9] Morandi A , Zambon A , Di Santo SG , et al. Understanding factors associated with psychomotor subtypes of delirium in older inpatients with dementia. J Am Med Dir Assoc. 2020;21(4):486‐492. 10.1016/j.jamda.2020.02.013 32241566

[10] Romero JP , Benito‐León J , Louis ED , Bermejo‐Pareja F . Under reporting of dementia deaths on death certificates: a systematic review of population‐based cohort studies. J Alzheimer's Dis. 2014;41:213‐221. 10.3233/jad-132765 24583403

[11] Villarejo A , Benito‐León J , Trincado R , et al. Dementia‐associated mortality at thirteen years in the NEDICES cohort study. J Alzheimer's Dis. 2011;26:543‐551. 10.3233/jad-2011-110443 21694455

[12] Kim YI , Kim YY , Yoon JL , et al. Cohort profile: national health insurance service‐senior (NHIS‐senior) cohort in Korea. BMJ Open. 2019;9:e024344. 10.1136/bmjopen-2018-024344 PMC6615810 31289051

[13] Kimm H , Yun JE , Lee S‐H , Jang Y , Jee SH . Validity of the diagnosis of acute myocardial infarction in Korean national medical health insurance claims data: the Korean heart study (1). Korean Circ J. 2012;42:10‐15. 22363378 10.4070/kcj.2012.42.1.10 PMC3283749

[14] Kim D , Yang PS , Yu HT , et al. Risk of dementia in stroke‐free patients diagnosed with atrial fibrillation: data from a population‐based cohort. Eur Heart J. 2019;40:2313‐2323. 10.1093/eurheartj/ehz386 31212315

[15] Kim D , Yang PS , Lip GYH , Joung B . Atrial fibrillation increases the risk of early‐onset dementia in the general population: data from a population‐based cohort. J Clin Med. 2020;9(11):3665. 10.3390/jcm9113665 33202611 PMC7697737

[16] Andrade SE , Kahler KH , Frech F , Chan KA . Methods for evaluation of medication adherence and persistence using automated databases. Pharmacoepidemiol Drug Safety. 2006;15:565‐574. 10.1002/pds.1230 16514590

[17] Deckers K , Schievink SHJ , Rodriquez MMF , et al. Coronary heart disease and risk for cognitive impairment or dementia: systematic review and meta‐analysis. PLoS One. 2017;12:e0184244. 10.1371/journal.pone.0184244 28886155 PMC5590905

[18] Haring B , Leng X , Robinson J , et al. Cardiovascular disease and cognitive decline in postmenopausal women: results from the women's health initiative memory study. J Am Heart Assoc. 2013;2:e000369. 10.1161/JAHA.113.000369 24351701 PMC3886762

[19] Friberg L , Andersson T , Rosenqvist M . Less dementia and stroke in low‐risk patients with atrial fibrillation taking oral anticoagulation. Eur Heart J. 2019;40:2327‐2335. 10.1093/eurheartj/ehz304 31095295 PMC6642728

[20] Lee JW , Choi EA , Kim YS , et al. Statin exposure and the risk of dementia in individuals with hypercholesterolaemia. J Intern Med. 2020;288:689‐698. 10.1111/joim.13134 32583471

[21] Swiger KJ , Manalac RJ , Blumenthal RS , Blaha MJ , Martin SS . Statins and cognition: a systematic review and meta‐analysis of short‐ and long‐term cognitive effects. Mayo Clin Proc. 2013;88:1213‐1221. 10.1016/j.mayocp.2013.07.013 24095248

[22] Evans MA , Golomb BA . Statin‐associated adverse cognitive effects: survey results from 171 patients. Pharmacotherapy. 2009;29:800‐811. 10.1592/phco.29.7.800 19558254

[23] Roy S , Hyman D , Ayyala S , et al. Cognitive function assessment in patients on moderate‐ or high‐intensity statin therapy. J Clin Med Res. 2020;12:255‐265. 10.14740/jocmr4144 32362974 PMC7188372

[24] Pujades‐Rodriguez M , Assi V , Gonzalez‐Izquierdo A , et al. The diagnosis, burden and prognosis of dementia: a record‐linkage cohort study in England. PLoS One. 2018;13:e0199026. 10.1371/journal.pone.0199026 29944675 PMC6019102

[25] Beydoun MA , Beydoun HA , Gamaldo AA , Teel A , Zonderman AB , Wang Y . Epidemiologic studies of modifiable factors associated with cognition and dementia: systematic review and meta‐analysis. BMC Public Health. 2014;14:643. 10.1186/1471-2458-14-643 24962204 PMC4099157

[26] Hugo J , Ganguli M . Dementia and cognitive impairment. Clin Geriatr Med. 2014;30:421‐442. 10.1016/j.cger.2014.04.001 25037289 PMC4104432

[27] Barnes DE , Yaffe K . The projected effect of risk factor reduction on Alzheimer's disease prevalence. Lancet Neurol. 2011;10:819‐828. 10.1016/s1474-4422(11)70072-2 21775213 PMC3647614

[28] McDowell I , Xi G , Lindsay J , Tierney M . Mapping the connections between education and dementia. J Clin Exp Neuropsychol. 2007;29:127‐141. 10.1080/13803390600582420 17365248

[29] Schneider LS , Mangialasche F , Andreasen N , et al. Clinical trials and late‐stage drug development for Alzheimer's disease: an appraisal from 1984 to 2014. J Intern Med. 2014;275:251‐283. 10.1111/joim.12191 24605808 PMC3956752

[30] Brodaty H , Seeher K , Gibson L . Dementia time to death: a systematic literature review on survival time and years of life lost in people with dementia. Int Psychogeriatr. 2012;24:1034‐1045. 10.1017/s1041610211002924 22325331

[31] Lee H , Park JH , Floyd JS , Park S , Kim HC . Combined effect of income and medication adherence on mortality in newly treated hypertension: nationwide study of 16 million person‐years. J Am Heart Assoc. 2019;8:e013148. 10.1161/jaha.119.013148 31422733 PMC6759906

[32] Rasmussen JN , Chong A , Alter DA . Relationship between adherence to evidence‐based pharmacotherapy and long‐term mortality after acute myocardial infarction. JAMA. 2007;297:177‐186. 10.1001/jama.297.2.177 17213401

