# Supplementary material for: Impact of dementia and drug compliance on patients with acute myocardial infarction
Source: Clin Cardiol. 2023 Jul 24;46(10):1253–9. doi: 10.1002/clc.24091 (PMC10577568; doi:10.1002/clc.24091)
Supplement: Supplementary file 1 — Figure S1. Kaplan‐Meier curves for comparison of AMI patients according to the presence of dementia and MPR of standard medications. Note: AMI = acute myocardial infarction, MPR = medication possession ratio, CCB = calcium channel blocker, ACE = angiotensin converting enzyme inhibitors, ARB = angiotensin receptor blockers, P2Y12 = P2Y12 inhibitors. [file CLC-46-1253-s001.pptx]

## Slide 1
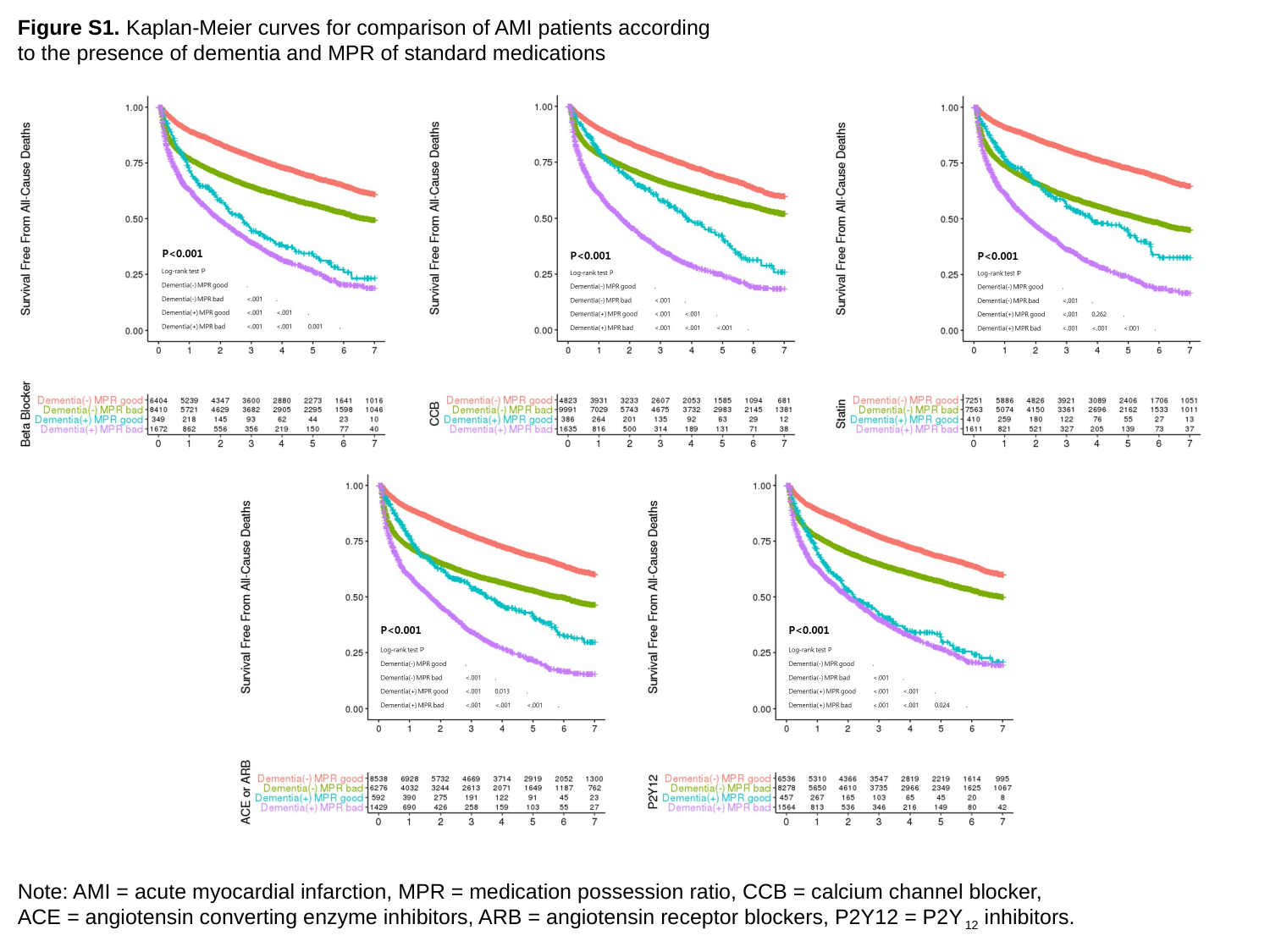

Figure S1. Kaplan-Meier curves for comparison of AMI patients according
to the presence of dementia and MPR of standard medications
Note: AMI = acute myocardial infarction, MPR = medication possession ratio, CCB = calcium channel blocker,
ACE = angiotensin converting enzyme inhibitors, ARB = angiotensin receptor blockers, P2Y12 = P2Y12 inhibitors.
